# Selection on tropane alkaloids in native and non‐native populations of *Datura stramonium*

**DOI:** 10.1002/ece3.5520

**Published:** 2019-08-23

**Authors:** Guillermo Castillo, Adriana Calahorra‐Oliart, Juan Núñez‐Farfán, Pedro L. Valverde, Juan Arroyo, Laura L. Cruz, Rosalinda Tapia‐López

**Affiliations:** ^1^ Departamento de Ecología Evolutiva Instituto de Ecología Universidad Nacional Autónoma de México (UNAM) Mexico City Mexico; ^2^ Departamento de Biología Universidad Autónoma Metropolitana‐Iztapalapa Mexico City Mexico; ^3^ Departamento de Biología Vegetal y Ecología Universidad de Sevilla Sevilla Spain; ^4^ Present address: Facultad de Enología y Gastronomía Universidad Autónoma de Baja California Baja California México

**Keywords:** biotic interactions, *Datura stramonium*, invasive species, selection of plant defence, tropane alkaloids

## Abstract

Theories of plant invasion based on enemy release in a new range assume that selection exerted by specialist herbivores on defence traits should be reduced, absent, or even selected against in the new environment. Here, we measured phenotypic selection on atropine and scopolamine concentration of *Datura stramonium* in eight native (Mexico) and 14 non‐native (Spain) populations. Native populations produced between 20 and 40 times more alkaloid than non‐native populations (atropine: 2.0171 vs. 0.0458 mg/g; scopolamine: 1.004 vs. 0.0488 mg/g, respectively). Selection on alkaloids was negative for atropine and positive for scopolamine concentration in both ranges. However, the effect sizes of selection gradients were only significant in the native range. Our results support the assumption that the reduction of plant defence in the absence of the plant's natural enemies in invasive ranges is driven by natural selection.

## Graphical abstract

Here, we measured phenotypic selection on two chemical defense against herbivores (atropine and scopolamine) in native (Mexico) and non‐native (Spain) populations of *Datura stramonium*. Our results support the assumption that the reduction in plant defense in the absence of the plant's natural enemies in invasive ranges is driven by natural selection.

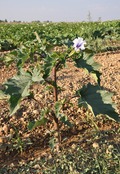

## 1 INTRODUCTION

Why some species succeed while others fail during invasion of novel environments is a topic in ecology and evolution with a long history that continues to be relevant today (Crawley, 1987; Elton, 1958; Sax et al., 2007). Hypotheses that have been proposed to explain the success of exotic species include changes in life history characteristics of invaders, the absence of natural enemies in the new habitat, the biological properties of communities, and/or environmental factors that render some ecosystems more prone than others to invasion by alien species (van Kleunen, Bossdorf, & Dawson, 2018; Maron & Vilà, 2001).

In alien species, it has been proposed that the factors that control population growth in their native habitat are absent or attenuated in novel environments (Chun, Kleunen, & Dawson, 2010; Elton, 1958). This includes their natural enemies (i.e., competitors, herbivores, pathogens), whose absence might release a plant population from checks on growth and enable them to outcompete local species (Enemy Release Hypothesis, ERH; Keane & Crawley, 2002; van Kleunen et al., 2018; Maron & Vilà, 2001). Populations released from pest pressure in a new environment may consequently allocate resources to fitness‐enhancing traits that in their native environment were invested in avoiding, resist, or diminishing damage by natural enemies. The resources “saved” from defence could possibly be reallocated to traits that increase plants' competitive ability in the new environment (Evolution of increased competitive ability, EICA; Blossey & Nötzold, 1995). Plants may not reduce their investment in defence but rather shift their strategy toward qualitative defences, for instance, low‐cost chemicals that function against generalist herbivores in the new habitat (Shift in defence hypothesis, SDH; Doorduin & Vrieling, 2011; Joshi & Vrieling, 2005; Müller‐Schärer, Schaffner, & Steinger, 2004). This shift in defence in the new habitat is expected to increase qualitative defence and decrease quantitative defence in the invaded range (Joshi & Vrieling, 2005), where qualitative defence can emerge as a novel weapon for defence against local herbivores that have not coevolved with the plant species (Novel weapons defence hypothesis, NWH; Callaway & Ridenour, 2004).

Evolution can be truly rapid during invasion (Whitney & Gabler, 2008) and adaptive (Blair & Wolfe, 2004; Maron, Vilà, Bommarco, Elmendorf, & Beardsley, 2004; Wolfe, Elzinga, & Biere, 2004). However, few studies have demonstrated genetic variance in trait that improves invasiveness (i.e., emergence time, tillering rate and biomass as documented in the perennial grass *Phalaris arundinacea*; Lavergne & Molofsky, 2007). Hence, genetic variance in plants' investment in defence in the invasive range can promote the adaptive evolution of lower levels of defence (e.g., Maron et al., 2004; Meyer, Clare, & Weber, 2005; Wolfe et al., 2004). The reduction of investment in defence by the invaders alone can constitute an advantage in a new environment free of natural enemies (Colautti, Ricciardi, Grigorovich, & MacIsaac, 2004). This advantage can be further augmented if such resources are allocated to increase plants' competitive ability (Colautti et al., 2004). Thus, defence trait evolution in a new habitat, free of natural enemies, is expected to be different than in the native environment. Specifically, a reduction in selective pressure exerted by plants' natural enemies in the new environment would select for a reduction in defence. Hence, contrasting selection on defensive traits would be expected between native and non‐native populations. This is the question we pursued in this study.

According to these hypotheses, native and non‐native populations of plant species may diverge in their defence trait values. Empirical evidence of evolutionary changes in plant defence between invasive and native populations of a species is mixed (Chun et al., 2010; Colautti et al., 2004; Inderjit, 2012; Müller, 2018; Rotter & Holeski, 2018). For instance, a recent meta‐analysis indicated that plant fitness‐related traits and generalist herbivores performance are higher in non‐native than native populations of plants, in accordance with theoretical expectations; however, plant defence traits (phytochemicals, physical defence) are also higher in the non‐native range, contradicting the hypothesis (Rotter & Holeski, 2018).

Evidence of changes in defence between native and non‐native populations of a species is increasing but does not offer strong support of predicted evolutionary outcomes. Another recent meta‐analysis assessed whether the predicted direction of change of chemical defence production in introduced and native ranges agrees with one hypothesis (i.e., EICA, SDH) (Müller, 2018). In nearly half of the tests, native and non‐native populations did not differ in the amount of chemical defence (see table 12.1 in Müller, 2018). However, the SDH classifies chemical defence as qualitative (alkaloids, glucosinolates, etc.) and quantitative (tannins, proteinases, etc.) due to their putative costs, and predicts higher investment in qualitative defence in the introduced range to confront generalist herbivores (since the specialists are absent) in contrast with EICA. Both SDH and EICA predict a reduction of investment in quantitative defence in the introduced range due to their costs and the absence of specialized herbivores. Overall, 40% of the tests (28/68) support the prediction of the EICA, while 56% of the tests (38/68) agree with the SDH (Müller, 2018).

A central aspect of hypotheses of plant invasion, however, is that the release of enemies in the non‐native environment should change selective pressure on the defensive characters of non‐native plants, hence promoting phenotypic divergence. To date, few studies have assessed this aspect (Colautti & Lau, 2015; Franks, Pratt, Dray, & Simms, 2008; van Kleunen et al., 2018). Accordingly, we measured the direction and magnitude of phenotypic selection on chemical defence in native and non‐native populations of the annual plant *Datura stramonium* L. (Solanaceae). While it is native to Mexico (Symon & Haegi, 1991), *D. stramonium* currently has a worldwide distribution (van Kleunen, Fischer, & Johnson, 2007; Weaver & Warwick, 1984). This and other species of *Datura* have been used as medicinal plants since pre‐Columbian times by Mexican cultures (De la Cruz, 1552; De Sahagún, 1577). Soon after the Conquest of Mexico in the XVth century, the plant was brought to Spain (Sanz‐Elorza, Dana‐Sánchez, & Sobrino‐Vespertinas, 2004), probably starting its invasion in Andalusia due to the concentration of trade routes there. Thus, approximately 500 generations might have passed since its original introduction into Europe, though it is likely that repeated introductions might have also occurred. Currently, *D. stramonium* has a wide distribution on the Iberian Peninsula (Sanz‐Elorza et al., 2004) and in France (J. Arroyo and J. Núñez‐Farfán, pers. obs. 2016). Previous evidence of the same native (except Joquicingo) and non‐native populations of *D. stramonium* used in this study shows that native and non‐native ranges differ markedly in average damage by herbivores (mean ± *SE*: 50.3 ± 1.3% vs. 2.5 ± 1.02% respectively; Valverde et al., 2015). Furthermore, *Datura* species are well known for their production of tropane alkaloids (Evans, 1979; Leete, 1959). Alkaloids are toxic to animals, affecting enzymes and altering different physiological processes; some have strong effects on the nervous systems (Mithöfer & Boland, 2012). Studies have shown selection on one or two tropane alkaloids of *D. stramonium* when consumed by dietary specialist or generalist herbivores (Castillo et al., 2014; Miranda‐Pérez et al., 2016; Shonle & Bergelson, 2000).

The aim of this study was to evaluate the variation among populations in the production of two major tropane alkaloids, atropine and scopolamine, by *D. stramonium* from the native and non‐native range. Since tropane alkaloids have been implicated as components of resistance to herbivores in this plant species (Castillo et al., 2014; Shonle & Bergelson, 2000), we expected that plants in the non‐native range (i.e., Spain) would show lower levels of this chemical defence relative to plants from the original distribution area (i.e., Mexico) due to the absence of specialized herbivores. Also, we assessed whether individual variation in both tropane alkaloids (i.e., atropine and scopolamine) is selected in both ranges. To date, few studies have measured selection on defence characters in native and non‐native ranges, a necessary assumption of most hypotheses based on enemy release.

## 2 MATERIALS AND METHODS

### 2.1 Study species

**Figure 1:**
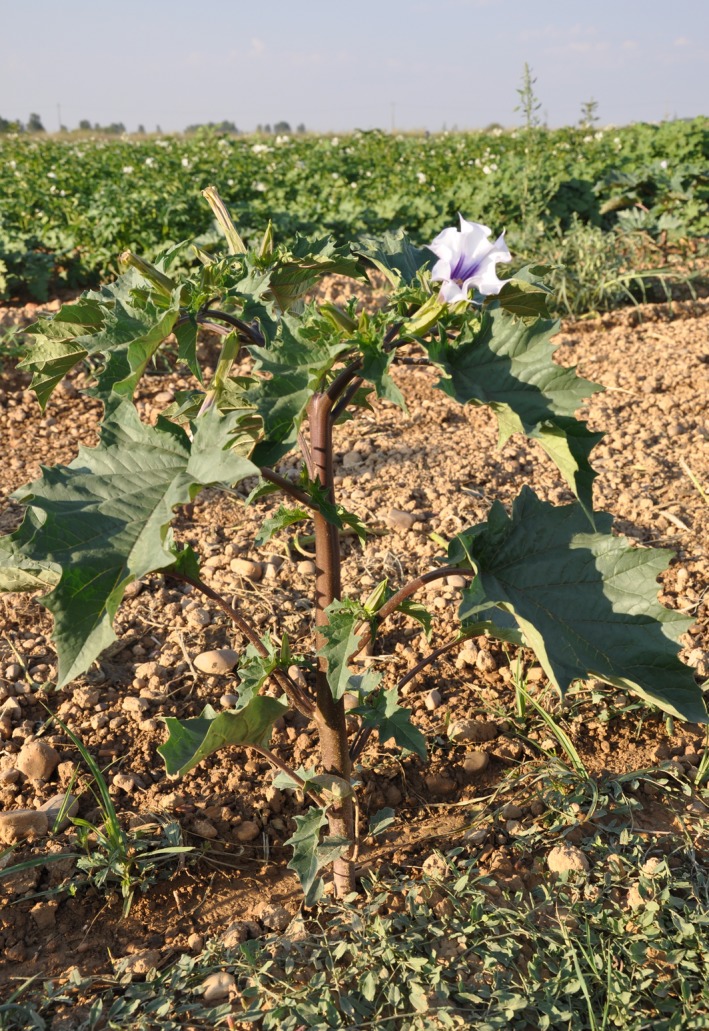
Reproductive plant of *Datura stramonium* in a cultivated field in the non‐native range (Spain)

*Datura stramonium* (Solanaceae) is a robust annual plant native to Mexico (Symon & Haegi, 1991) but which is currently widely distributed worldwide (Weaver & Warwick, 1984; Figure 1). In its native region, it is a summer annual that grows in open, disturbed places along roadsides, on the edges of cultivated land and along streams (Núñez‐Farfán, 1995). In Mexico, it is most common in temperate sites (Castillo et al., 2013) along the Trans‐Mexican Volcanic Belt that runs between the Pacific Ocean to the Gulf of Mexico in central Mexico (Ferrari, Orozco‐Esquivel, Manea, & Manea, 2012). Although it can be preyed upon by generalist herbivores (e.g., *Sphenarium* spp. Orthoptera: Pyrgomorphidae), most damage is caused by specialist leaf herbivores—species of the genus *Epitrix* as well as *Lema trilineata daturaphila* (Coleoptera: Chrysomelidae) (Núñez‐Farfán & Dirzo, 1994). Weevils of the genus *Trichobaris* (Coleoptera: Curculionidae) are specialized seed predators of *Datura* (De‐la‐Mora et al., 2018). *Trichobaris soror* is tightly associated with *D. stramonium* along the Trans‐Mexican Volcanic Belt (De‐la‐Mora, Piñero, & Núñez‐Farfán, 2015) and exerts selection on plants' scopolamine concentration (Miranda‐Pérez et al., 2016).

*Datura stramonium* is widely distributed on the Iberian Peninsula, occurring in cultivated fields, ruderal habitats like roadsides, riverbanks, and plowed fields, among others. It is a problematic species that merits biological control (Sanz‐Elorza et al., 2004). A previous study showed that, in southern Spain, *D. stramonium* is mainly damaged by the dietary generalist herbivore *Helicoverpa armigera* (Lepidoptera: Noctuidae) which feeds upon the leaves but also consumes developing seeds, until pupation (Valverde et al., 2015).

### 2.2 Sampled populations

Populations of *D. stramonium* were sampled in 2010 and 2011. Fourteen and eight populations of Spain and Mexico, respectively, were sampled (Table 1). Linear distances between populations ranged from 5 to 468 km in Spain, and from 20 to 300 km in Mexico. The sampled populations occurred within different plant communities (Table 1). In each population, we sampled 8–33 randomly selected reproductive individual plants (Table 1). In order to determine the average concentration of leaf tropane alkaloids, atropine and scopolamine, we collected a random sample of 8–40 leaves per plant in each population, and all fruits produced as a proxy of individual fitness (Castillo et al., 2014; Valverde et al., 2015).

**Table 1:** Geographic location of populations of *Datura stramonium* sampled in the native (Mexico) and non‐native (Spain) ranges. Sample size is provided

Range	Population	Province	Geographic position	*N*
Native	Acolman	Estado de México	19°41′13.56″N 98°50′06.72″W	28
Native	Joquicingo	Estado de México	19°06′55.08″N 99°31′05.16″W	30
Native	Patria Nueva	Hidalgo	20°22′35.40″N 99°02′53.16″W	30
Native	San Martìn	Estado de México	19°18′17.28″N 98°28′52.28″W	25
Native	Sanabria	Michoacán	19°34′37.20″N 101°34′12.00″W	32
Native	Santo Domingo	Morelos	19°00′42.48″N 99°03′45.72″W	25
Native	Tzin Tzun Tzan	Michoacán	19°38′10.32″N 101°35′32.64″W	30
Native	Valsequillo	Puebla	18°57′00.00″N 98°10′49.08″W	33
Non‐native	Bolonia	Cádiz	36°05′09.99″N 05°46′07.57″W	30
Non‐native	Cabeza La Vaca	Badajoz	38°06′48.00″N 06°24′23.36″W	29
Non‐native	Cardeña	Córdoba	38°14′56.63″N 04°12′58.45″W	30
Non‐native	Castañuelos	Huelva	37°56′19.83″N 06°35′02.97″W	8
Non‐native	Don Fadrique	Granada	37°57′39.75″N 02°26′08.75″W	23
Non‐native	El Pedroso	Sevilla	37°50′12.81″N 05°45′58.67″W	29
Non‐native	Gerena	Sevilla	37°31′28.86″N 06°11′24.76″W	13
Non‐native	El Higueral	Almería	37°23′12.61″N 02°29′56.48″W	29
Non‐native	Hinojos 1	Huelva	37°18′00.39″N 06°22′41.72″W	16
Non‐native	Hinojos 2	Huelva	37°19′28.36″N 06°25′32.45″W	21
Non‐native	Lora del Río	Sevilla	37°39′33.28″N 05°32′05.93″W	29
Non‐native	Pinilla	Murcia	37°41′03.10″N 01°17′00.62″W	22
Non‐native	Valdeflores	Sevilla	37°43′02.23″N 06°18′50.44″W	28
Non‐native	Zubia	Granada	37°07′47.28″N 03°35′57.06″W	29

### 2.3 Tropane alkaloid concentration

We used high‐performance liquid chromatography (HPLC) to quantify the concentration of atropine and scopolamine in leaves of *D. stramonium* (El Bazaoui, Bellimam, & Soulaymani, 2011). The extraction method consisted of a series of acid–base reactions (see Castillo et al., 2013, 2014). The samples were injected into a Waters Alliance 2695 HPLC device. We used a reverse‐phase column (Discovery C‐18 Supelco Analytical) at 30 μC. The injection volume was 30 ml with a flow rate of 1 ml/min. The mobile phase was a solution of acetonitrile, methanol, and a 30 mM phosphate buffer at a pH of 6.00 (9:6.9:84.1, v/v/v). The Diode Array HPLC detector used a wavelength of 210 nm. The curves obtained from each sample were compared against a standard solution of atropine and scopolamine (1 mg/ml; Sigma‐Aldrich Laboratories) and the mean alkaloid concentration (mg/g) per population was estimated.

### 2.4 Phenotypic variation of tropane alkaloids concentration

To test the effect of geographic range (Spain, Mexico) and population within range on atropine and scopolamine concentrations, we conducted two nested analyzes of variance (ANOVA). Population nested within range was declared as a random effect. In order to meet normality, alkaloid concentrations were transformed to ln(*x* + 1).

### 2.5 Phenotypic selection gradients

We evaluated whether the relationship between atropine and scopolamine concentration with plant fitness was significant in each native and non‐native population (data available at Castillo et al. 2019, https://doi.org/10.5061/dryad.j656q74). Standardized partial linear selection gradients (β*_i_*; Lande & Arnold, 1983) were obtained by fitting multiple linear regressions of relative plant fitness as a function of the concentration of the two defence tropane alkaloids in each population. Phenotypic values of defence traits were standardized ($\bar{x}$ = 0, *s* ^2^ = 1) prior to analysis. Individual fitness was relativized (*w_i_* = *x_i_*/$\bar{x}$), where *x_i_* is the total number of fruits produced per plant and $\bar{x}$ is the population's average number of fruits per plant. Nested analyzes of variance and multiple regression analyzes were performed using the JMP statistical package (v. 9.0; SAS Institute).

### 2.6 Effect size of selection on defence

We performed a meta‐analysis to estimate the effect size of selection gradients on atropine and scopolamine concentration between the native and non‐native ranges. We used the effect size, since it is a statistical parameter that allows us to compare, on the same scale, the results from different analyzes, that share a common effect of interest, phenotypic selection gradients between ranges in this case (Koricheva, Gurevitch, & Mengersen, 2013). Because the selection gradients were obtained from multiple regression models with the same covariance structure, slopes are reliable metrics to estimate effect sizes (Becker & Wu, 2007). Effect size of each alkaloid between ranges was estimated using the standardized selection gradient and its corresponding variance (Borenstein, Hedges, Higgins, & Rothstein, 2009; Ellis, 2010). Mean effect size and its corresponding 95% confidence interval (CI) were obtained for each tropane alkaloid. If the confidence interval did not include zero, we inferred a significant effect of intensity and/or direction of selection in the native or non‐native range. The meta‐analyzes were performed using the metaphor package (Viechtbauer, 2010) for R v3.0.2 software (R Core Team, 2011).

## 3 RESULTS

**Figure 2:**
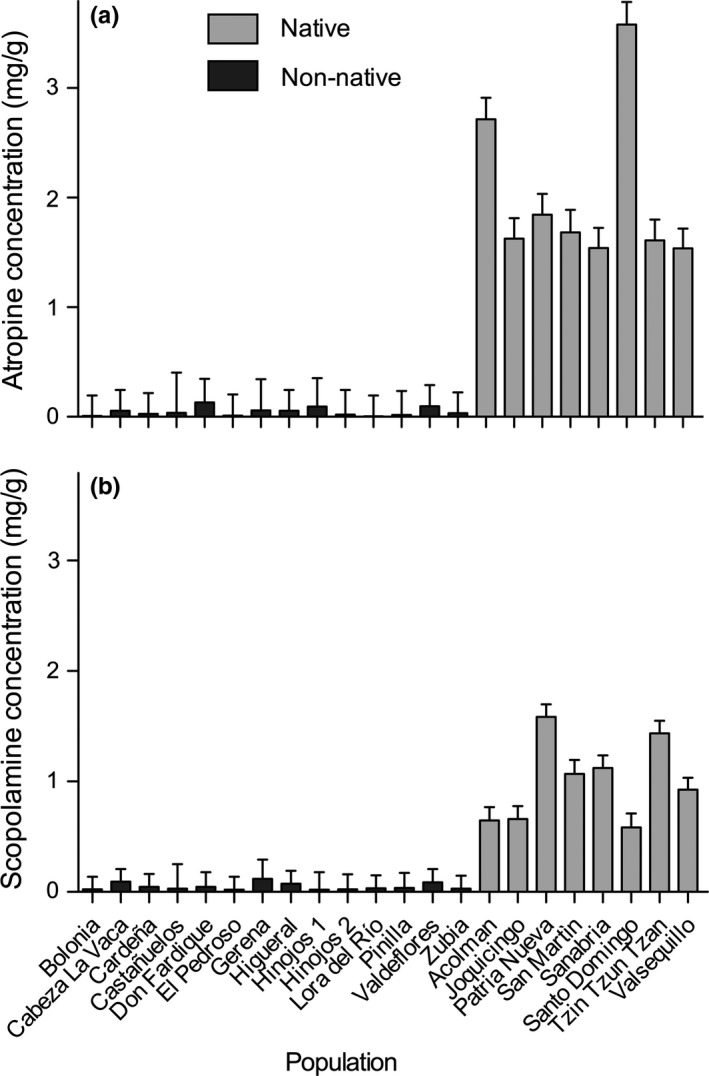
Average (+*SE*) concentration of atropine (a) and scopolamine (b) in native (Mexico) and non‐native (Spain) populations of *Datura stramonium*

Populations of *D. stramonium* from the native and non‐native ranges differed in their mean concentration of tropane alkaloids (Table 2). Native populations produced up 20 times more atropine and 40 times more scopolamine than non‐native populations (Figure 2). Most of the variance was explained by the range, but there was also a significant fraction of variance explained by the population term (12.6% and 10.8% for atropine and scopolamine, respectively).

**Table 2:** Nested analysis of variance of atropine (a) and scopolamine (b) concentration between geographical ranges (Mexico and Spain) and among populations within ranges

Source	SS	*df*	MS	*F*	*p*
(a) Atropine					
Range	112.605	1	112.605	282.93	<.0001
Population [Range]	8.066	20	0.4033	4.70	<.0001
Error	46.906	547	0.0857		
(b) Scopolamine					
Range	39.260	1	39.260	145.75	<.0001
Population [Range]	5.457	20	0.272	4.12	<.0001
Error	36.150	547	0.066		

Note

(a) *R* ^2^_adj._ = 0.718; (b) *R* ^2^_adj._ = 0.555.

The analysis of the relationship between relative plant fitness and alkaloid concentration indicated only a few significant selection gradients (Table 3). Atropine concentration was selected in six populations, three in the native and three in the non‐native range; in all cases it was selected against (Table 3). In contrast, scopolamine concentration was positively selected in three populations in the native range and only in one population in the non‐native range (Table 3).

**Table 3:** Standardized selection gradients (*β_i_*) of atropine and scopolamine concentration in native (Mexico) and non‐native (Spain) populations of *Datura stramonium*

Range	Population	*N*	Defensive trait	*β*	*SE*	*t*	*p*
Mexico	Acolman	morerows="1">28	**Atropine**	**−0.816**	**0.312**	**−2.61**	**.0149**
		Scopolamine	0.576	0.312	1.85	.0767
	Joquicingo	morerows="1">30	Atropine	−0.292	0.184	−1.58	.1257
		Scopolamine	−0.010	0.184	−0.05	.9579
	Patria Nueva	morerows="1">30	Atropine	−0.064	0.211	−0.30	.7642
		**Scopolamine**	**−0.424**	**0.211**	**−2.01**	**.0546**
	San Martín	morerows="1">25	Atropine	−0.447	0.230	−1.94	.0652
		Scopolamine	0.052	0.230	0.23	.8220
	Sanabria	morerows="1">32	Atropine	−0.167	0.227	−0.73	.4694
		Scopolamine	0.039	0.227	0.17	.8659
	Santo Domingo	morerows="1">25	**Atropine**	**−0.576**	**0.198**	**−2.91**	**.0082**
		**Scopolamine**	**0.668**	**0.198**	**3.37**	**.0028**
	Tzin Tzun Tzan	morerows="1">30	**Atropine**	**−0.523**	**0.221**	**−2.37**	**.0252**
		**Scopolamine**	**0.524**	**0.221**	**2.37**	**.0250**
	Valsequillo	morerows="1">33	Atropine	0.202	0.228	0.89	.3827
		Scopolamine	−0.051	0.228	−0.22	.8260
Spain	Bolonia	morerows="1">30	Atropine	−0.308	0.210	−1.46	.1549
		Scopolamine	0.309	0.210	1.47	.1537
	Cabeza La Vaca	morerows="1">29	Atropine	−0.252	0.213	−1.18	.2483
		Scopolamine	0.053	0.213	0.25	.8046
	Cardeña	morerows="1">30	Atropine	−0.075	0.246	−0.31	.7614
		Scopolamine	−0.097	0.246	−0.39	.6972
	Castañuelos	morerows="1">8	Atropine	−0.392	0.436	−0.90	.4094
		Scopolamine	0.019	0.436	0.04	.9664
	Don Fadrique	morerows="1">23	Atropine	−0.444	0.246	−1.81	.0860
		Scopolamine	−0.010	0.246	−0.04	.9684
	El Pedroso	morerows="1">29	**Atropine**	**−0.513**	**0.252**	**−2.04**	**.0520**
		Scopolamine	0.135	0.252	0.54	.5967
	Gerena	morerows="1">13	Atropine	−0.695	0.412	−1.69	.1227
		Scopolamine	0.712	0.412	1.73	.1151
	El Higueral	morerows="1">29	Atropine	−0.289	0.222	−1.30	.2039
		Scopolamine	0.029	0.222	0.13	.8977
	Hinojos 1	morerows="1">16	**Atropine**	**−0.972**	**0.287**	**3.38**	**.0049**
		**Scopolamine**	**0.707**	**0.287**	**2.46**	**.0286**
	Hinojos 2	morerows="1">21	Atropine	−0.414	0.386	−1.07	.2979
		Scopolamine	0.436	0.386	1.13	.2731
	Lora del Río	morerows="1">29	Atropine	−0.221	0.213	−1.04	.3101
		Scopolamine	0.020	0.213	0.09	.9254
	Pinilla	morerows="1">22	Atropine	0.039	0.343	0.11	.9105
		Scopolamine	0.374	0.343	1.09	.2890
	Valdeflores	morerows="1">28	Atropine	−0.047	0.206	0.23	.8200
		Scopolamine	0.169	0.206	0.82	.4217
	Zubia	morerows="1">29	**Atropine**	**−0.564**	**0.233**	**−2.42**	**.0229**
		Scopolamine	0.263	0.233	1.13	.2698

Note

Significant values in bold type.

Abbreviation: *SE*, standard error.

**Figure 3:**
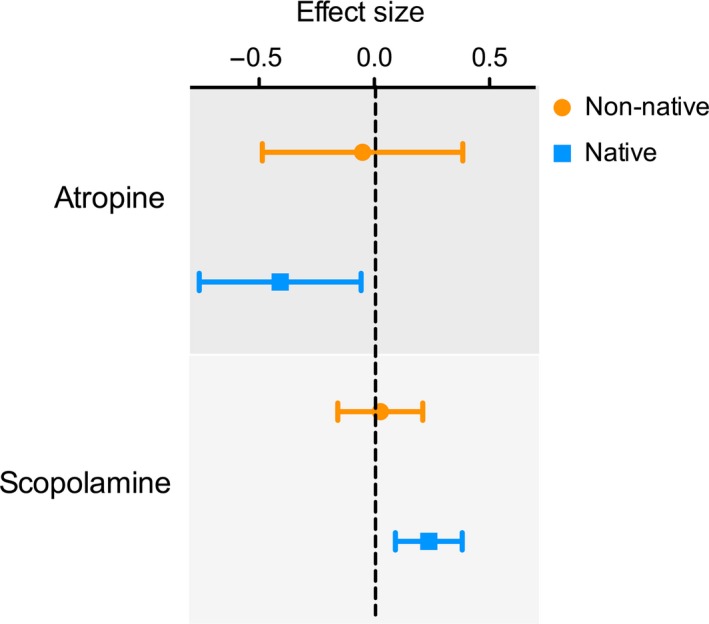
Effect size and 95% confidence interval of selection gradients of atropine and scopolamine concentration in populations of *Datura stramonium* in the native (Mexico) and non‐native (Spain) ranges

The meta‐analysis indicated that the average effect size of selection gradients of atropine was −0.394 (CI: −0.712, −0.076) and −0.063 (CI: −0.467, 0.341) in the native and non‐native ranges, respectively (Figure 3). The CI of the native range did not overlap zero, while the average value of non‐native plants was undistinguishable from zero. The effect size of selection on scopolamine concentration was positive for both ranges (native, 0.204 [CI: 0.068, 0.339] and 0.059 non‐native [CI: −0.117, 0.236]) but significant only for the native range (Figure 3).

## 4 DISCUSSION

The relaxation of selective pressure on plant defensive characters in the non‐native relative to native ranges is central to hypotheses that propose that plants' invasion of new ranges is promoted by the absence of their natural enemies. Here, we found that in the introduced range (Spain), populations of *D. stramonium* produced much less tropane alkaloid concentration than populations in the native range (Mexico), consistent with these hypotheses. Although selection on tropane alkaloids was unexpectedly in the same direction in both ranges, the average effect size of selection gradients of atropine and scopolamine concentration was not different from zero in the non‐native range but significant in the native range. Thus, relaxation of selection on defence in the non‐native range supports theoretical predictions.

It has been stressed that evolutionary studies of plant invasion should study natural selection and adaptation in both the native and introduced ranges (Colautti & Lau, 2015; van Kleunen et al., 2018). In relation to this, theories of plant invasion based on enemy release in the introduced range assume that selection exerted by specialist herbivores on defence traits would be reduced, absent, or even negative in the introduced environment (Blossey & Nötzold, 1995; Keane & Crawley, 2002; Maron & Vilà, 2001). Defence traits might be maintained in populations in the introduced environments if they are not costly (i.e., neutral variation), but if the investment in the production of defence traits is costly, resources are expected to be reallocated to increase competitive ability (EICA; Blossey & Nötzold, 1995). In that case, selection against defence is expected. On the contrary, according to SDH, selection favouring an increase of qualitative defence and a decrease of quantitative defence are expected in the introduced range to confront generalist herbivores. Our results, in general, point a reduction in plant defence in the introduced range, since the amount of atropine and scopolamine concentration is 20–40 times lower than in the native range. Such a reduction of qualitative defence is not expected under the SDH.

Selection against atropine concentration in three populations in the introduced range and in three of the native populations points out the nondefensive role of this alkaloid, perhaps because it is used by herbivores as a cue to locate the host plants, or worse, because it may act as a phagostimulant (Castillo et al., 2014). On the other hand, scopolamine concentration was positively selected in one non‐native population suggesting that even at low levels it may enhance fitness in a range where specialist herbivores of *D. stramonium* are absent. However, whether low levels of damage in the non‐native range are due to high effectiveness of alkaloids as anti‐herbivore defence and/or to low pressure by herbivores (i.e., low abundance) remains to be further studied. Available evidence in the same system and ranges points in the direction of the latter (see Valverde et al., 2015).

In the native range, scopolamine was positively selected in three populations. Previous evidence shows that this alkaloid is a component of resistance to herbivores of *D. stramonium* in its native range, though it does not account for all variation in resistance owing to spatial variation in the pressure exerted by specialist herbivores (see Castillo et al., 2014; Miranda‐Pérez et al., 2016). For instance, the dietary specialist leaf beetle *Lema trilineata daturaphila* and the seed predator *Trichobaris soror* are absent in tropical, low altitude, locations of central Mexico (Castillo et al., 2014; J. Núñez‐Farfán, pers. obs.), and thus defence traits are not selected in some locations, just as is expected in the non‐native range. However, in the native range, the levels of plant damage by herbivores and defence are often high (Castillo et al., 2013). This heterogeneity of herbivores and defence in the native range must be taken into account when comparing native versus introduced populations, since the source populations are often an unknown subset of all native range populations, as stressed by Colautti et al. (2004). Unfortunately, the native populations of *D. stramonium* that was the source introduced into Spain remains to be determined.

Alkaloids, like other secondary compounds (i.e., glucosinolates), are considered qualitative defence traits that may function against generalist herbivores and are thus expected to increase in a new range (Joshi & Vrieling, 2005). Our results do not support this prediction, since Spanish populations of *D. stramonium* had the lowest values of atropine and scopolamine. Previous evidence indicated that populations of *D. stramonium* in the non‐native range (Spain) have low levels of damage (ca. 2.5% of leaf area lost) by different generalist herbivores but mainly by the moth *H. armigera* (Valverde et al., 2015). Why are levels of plant damage low in the non‐native range? At least two explanations can be offered. First, the low concentration of both tropane alkaloids might be enough to prevent damage by local herbivores that have not coevolved with *D. stramonium*. Second, the low levels of herbivory in the non‐native range may obey to the low pressure exerted by generalist herbivorous insects. We found no support for the first explanation since our meta‐analysis revealed that tropane alkaloids have no selective value in the non‐native range. Our study suggests that the low load of herbivores in the populations of *D. stramonium* in southern Spain (Valverde et al., 2015) appears to be a plausible explanation.

Finally, further studies will help to expand the study of the evolution of plant defence in new ranges. For instance, we still need to assess, using experimental manipulations of herbivores: (a) the extent of genetic variation underlying plant chemistry and (b) the differences between these genotypes in fitness (van Kleunen et al., 2018; Rausher, 1996). Moreover, the amount of tropane alkaloids in tissues of *D. stramonium* does vary ontogenetically (El Bazaoui et al., 2011; Kariñho‐Betancourt, Agrawal, Halitschke, & Núñez‐Farfán, 2015), genetically, and in relation to physical environmental factors such as nitrogen level in the soil, temperature, water, and light incidence (Nowacki, Jurzyesta, & Gorski, 1975; Waller & Nowacki, 1978). Studies to determine the role of phenotypic plasticity on the evolution of plant defence between ranges would further increase our understanding of plant invasion (Franks et al., 2008).

## 5 CONCLUSION

Our phenotypic selection analysis of defensive tropane alkaloids in native and non‐native populations of *D. stramonium* supports the theoretical prediction, common to several hypotheses but rarely assessed, of a reduction in plant defence in the introduced range promoted by the absence of dietary specialist herbivores. Assuming that *D. stramonium* was introduced into Europe during the XVIth century, the reduction in alkaloid concentration might represent an evolutionary response to selection over almost 500 generations.

## CONFLICT OF INTEREST

None declared.

## AUTHOR CONTRIBUTIONS

JNF, PLV, JA, and GC designed the study; GC, PLV, JNF, LLC, and JA, carried out the fieldwork; GC, ACO, LLC, RTL carried out the laboratory work; GC, PLV, JNF, and JC did the statistical analyzes, JNF wrote the manuscript, and all authors revised, corrected, and approved the manuscript.

## Data Availability

Data available from the Dryad Digital Repository: https://doi.org/10.5061/dryad.j656q74.
